# Disseminated miliary tuberculosis with cutaneous involvement in a patient with HIV

**DOI:** 10.1590/0037-8682-0276-2023

**Published:** 2023-09-22

**Authors:** André de Almeida Vieira, Edson Marchiori, Roberto Mogami

**Affiliations:** 1 Universidade do Estado do Rio de Janeiro, Departamento de Medicina Interna, Disciplina de Radiologia, Rio de Janeiro, RJ, Brasil.; 2 Universidade Federal do Rio de Janeiro, Departamento de Radiologia, Rio de Janeiro, RJ, Brasil.

A 35-year-old man presented to the hospital with complaints of pain in his left elbow and right knee, persistent cough, weight loss, night sweats, and generalized weakness over the past few months. Recent episodes of recurrent fever and a noticeable decrease in appetite were reported. He was diagnosed with human immunodeficiency virus (HIV) infection 5 years ago and has been on antiretroviral therapy since then. Although his adherence to the therapy was suboptimal, with frequent missed doses, he had not experienced any major opportunistic infections or HIV-related complications.

**FIGURE 1:**
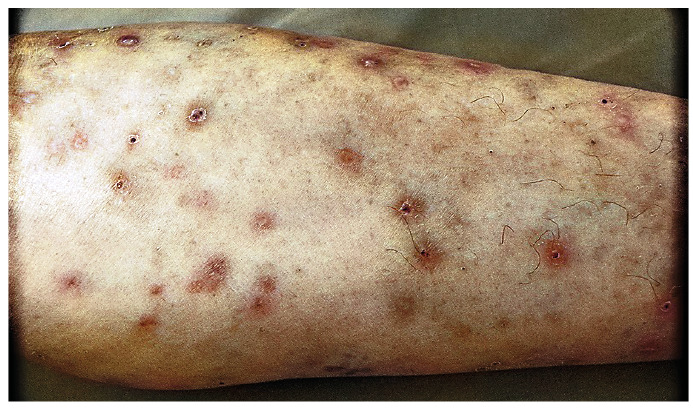
Right forearm with an erythematous papular rash.

**FIGURE 2:**
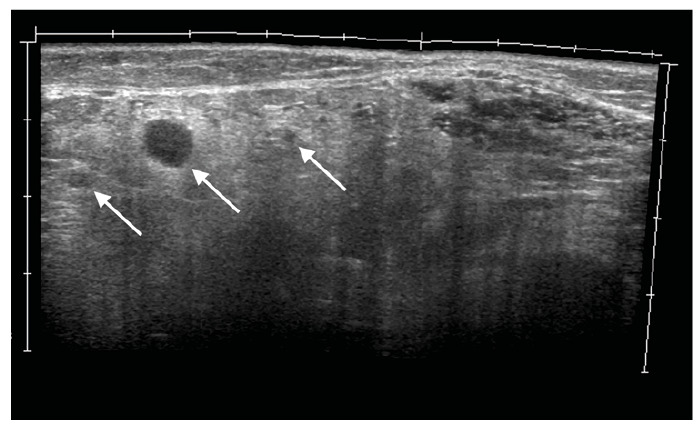
Ultrasound of the subcutaneous layer of the forearm displaying several granulomas (arrows).

**FIGURE 3:**
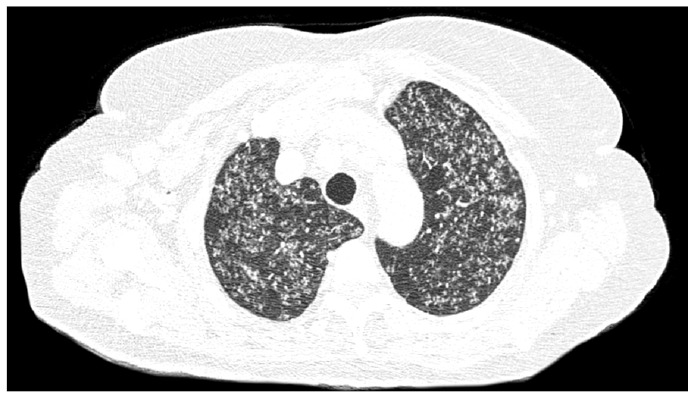
Chest computed tomography; lung window. Diffuse miliary nodules.

Skin examination revealed an erythematous papular rash (Figure 1). Ultrasound of the subcutaneous layer showed several hypoechoic nodules of varying sizes (Figure 2). Chest computed tomography showed several small hyperdense nodules compatible with miliary tuberculosis (Figure 3). Ultrasound of the elbow and knee revealed synovial thickening and effusion consistent with arthritis. 

Disseminated miliary tuberculosis is a severe form of tuberculosis resulting from *Mycobacterium tuberculosis* dissemination via the bloodstream, affecting multiple organs^1^. Several extrapulmonary manifestations may occur, including arthritis^2^ and a cutaneous form^3^.

The subcutaneous layer of the affected area may show ill-formed granulomas, which are aggregates of immune cells and necrosis, or micro-abscesses composed of neutrophils and acid-fast bacilli^3^. The diagnosis of disseminated miliary tuberculosis with cutaneous involvement requires a combination of clinical findings, microbiological tests, and imaging studies.
